# Uncertainty Propagation for NIST Visible Spectral Standards

**DOI:** 10.6028/jres.109.023

**Published:** 2004-06-01

**Authors:** James L. Gardner

**Affiliations:** CSIRO National Measurement Laboratory Lindfield, Australia 2070

**Keywords:** irradiance, radiometry, responsivity, uncertainty

## Abstract

Uncertainties in the NIST spectral standards for detectors and sources in the visible wavelength range are propagated from the high accuracy cryogenic radiometer measurements, taking correlations into account at every stage. Partial correlations between spectral values at different wavelengths, important for subsequent radiometric calculations, are estimated. Uncertainty propagation through fitting and through transfer spectral measurements is described in detail. Detector uncertainties are propagated through the spectral comparator facility for external calibrations and for internal photometric quantities. Uncertainties in spectral irradiance are derived for the detector-based temperature determination, then propagated through working standards to calibrated artifacts. Spectral irradiance calibrations are generally provided at a limited number of wavelengths. Interpolation, rather than fitting, is recommended for the interpolation of NIST-provided spectral irradiance values.

## 1. Introduction

Radiometric calibrations of spectral responsivity or spectral irradiance at particular wavelengths are required for a number of applications. Standards for these quantities are provided to external customers and used internally in deriving reference values for such quantities as color, correlated color temperature and distribution temperature. Most of the national laboratories provide such calibrations in spectral ranges from the ultra-violet to infrared regions. Primary standards in radiometry are increasingly detector-based, traced ultimately to power or irradiance measurements made with a cryogenic radiometer. While some laboratories operate such devices at the exit of a monochromator, or with laser-based sources tunable over a wide wavelength range, it is both time-consuming and expensive to cover all the wavelengths at which reference measurements may be required; most national metrology institutes provide measurements at a limited number of wavelengths and then apply either interpolation or fitting techniques to cover the complete range. New values generated from the limited set are then correlated through dependence on the common input set. A thorough propagation of uncertainties requires this correlation to be taken into account when spectral values are combined, a common practice in radiometry. Accurate estimate of the uncertainties resulting from the assumptions made in modeling the measurement and transfer process, rather than an over-estimate, increases the chance of detecting systematic components that may have been overlooked. The National Institute of Standards and Technology (NIST) methods and procedures in radiometry and photometry are described in detail in the Special Publication SP250 series [1–3]. Some of the descriptions of the traceability paths are now dated, and none provide an estimate of the partial correlations in the reported spectral quantities required for a modern uncertainty calculation when combining values in spectral integrals.

Standards for spectral quantities (spectral irradiance and spectral responsivity) at NIST are currently based on absolute detector measurements at a limited number of wavelengths. Spectral responsivity measurements are used to calibrate the response of a filter radiometer, in turn used to estimate the temperature of a black-body radiator and hence provide the reference standard for spectral irradiance measurements. These reference standards, both for detectors and sources, are transferred to working standards for external calibrations, and used internally in deriving reference standards for photometric and colorimetric measurements. Following a section on the basic principles, this paper considers uncertainty propagation through the spectral measurement chain at NIST, concentrating on the visible- and near-visible- wavelength ranges. Interpolation alternatives for users of NIST-provided values of spectral irradiance are discussed. The final section considers the alternatives of fitting and interpolation where tunable lasers are used to give effectively continuous coverage through the spectral range of interest.

## 2. Uncertainty Propagation

### 2.1 Basic Principles

Uncertainty propagation is described in detail in the *ISO Guide to the Expression of Uncertainty in Measurement* [4]. The uncertainty in a quantity *y* formed by combining *N* measured quantities *x_i_* through the relationship *y* = *f* (*x*_1_,*x*_2_, *x_N_*) is given by

$$u^{2}(y){\sum_{i=1}^{N}\left(\frac{\partial f}{\partial x_{i}}\right)}^{2}u^{2}(x_{i})+\sum_{i=1}^{N}\sum_{j\neq i=1}^{N}\frac{\partial f}{\partial x_{i}}\frac{\partial f}{\partial x_{j}}u(x_{i},x_{j}),$$ (1)

where *u*(*x_i_*) is the uncertainty in *x_i_* and *u*(*x_i_*, *x_j_*) is the covariance between *x_i_* and *x_j_*. For independent input quantities, the covariance between pairs of variables is zero and Eq. (1) reduces to the “sum of squares” commonly applied. The derivatives ∂*f*/∂*x_i_* are sensitivity coefficients for the dependence of *y* on the various measured quantities. Given that *u*^2^(*x_i_*) = *u*(*x_i_*, *x_i_*), Eq. (1) can be expressed as

$$u^{2}(y)={f_{x}}^{T}U_{x}f_{x},$$ (2)

where

$$f_{x}^{T}=\left(\frac{\partial f}{\partial x_{1}}\frac{\partial f}{\partial x_{2}}\cdot \cdot \frac{\partial f}{\partial x_{N}}\right)$$ (3)

is a row vector of sensitivity coefficients (superscript ^T^ indicates the transpose) and

$$U_{x}=\left[u(x_{i},x_{j})\right]$$ (4)

is the symmetric *N* × *N* variance-covariance matrix with variance of the input quantities (square of the uncertainty) in diagonal elements, and the covariance between input quantities elsewhere. Knowledge of the sensitivity coefficients and the variance-covariance matrix is sufficient to propagate the uncertainty to any output quantity that is formed by combining the input quantities. The covariance between any two quantities *y*_1_, *y*_2_ that depend on the same set of input quantities is found by including the two sensitivity vectors in Eq. (3), i.e.,

$$u(y_{1},y_{2})=f_{x}^{T}U_{x}g_{x}$$ (5)

where *y*_1_ = *f*(*x*_1_, *x*_2_,..*x_N_*) and *y*_2_ = *g*(*x*_1_, *x*_2_,..*x_N_*) have different functional dependencies on the input quantities. In the sections below, no distinction will be made between variance and covariance; variance is taken as the covariance of a quantity with itself.

It is often useful to describe correlations in terms of correlation coefficients. These are defined by

$$\begin{array}{l} r(y_{1},y_{2})=\frac{u(y_{1},y_{2})}{u(y_{1})u(y_{2})}\\ =\frac{u_{\text{rel}}(y_{1},y_{2})}{u_{\text{rel}}(y_{1})u_{\text{rel}}(y_{2})} \end{array},$$ (6)

where *u*_rel_(*y*_1_, *y*_2_) is the relative covariance, that is, the covariance *u*(*y*_1_, *y*_2_) divided by the product *y*_1_*y*_2_ and *u*_rel_(*y*_1_), *u*_rel_(*y*_2_) are the familiar relative uncertainties in *y*_1_, *y*_2_, respectively.

### 2.2 Fitting

Fitting is a two-stage process frequently applied to estimate a functional form that represents the relationship between a limited set of input quantities (one dependent, the other independent and usually representing wavelength in radiometry) and then to use this function to calculate the dependent value at other independent values. Uncertainty propagation through Eq. (5) is applied to each of these stages.

Suppose we have a set of *N* values *y_i_* each dependent on the wavelength *λ_i_* and the *P* parameters *a_j_* where the values *y_i_* are assumed to be represented by the function

$$y_{i}^{\prime }=f(a_{1}..a_{P};\lambda_{i}).$$ (7)

Estimates of the values of the parameters *a_j_* are found by least-squares minimization of

$$\chi^{2}={\sum_{i}\left(y_{i}^{\prime }-y_{i}\right)}^{2}/(N-P).$$ (8)

Once the parameter estimates are found, it is possible to calculate the sensitivity coefficients representing the dependence of each parameter *a_j_* on each of the input quantities *y_i_*, either numerically or by direct differentiation [5]. Hence the variance-covariance matrix ***U****_a_* = [*u*(*a_i_*, *a_j_*)] of the parameters (correlated through dependence on the common set of input quantities) is calculated through Eq. (5).

The minimization is improved by weighting the terms of Eq. (8) by the inverse of the variance of the input *y_i_* values. A complication here is that these input quantities may be correlated through systematic errors in the measurement process, or through a common dependence on other quantities. Commercial programs performing least-squares minimization can usually handle input values with differing uncertainties, but not those that may be correlated. Woeger [6] has described a technique that propagates uncertainties including input correlations, with Eq. (8) redefined as minimizing

$$\chi^{2}=M^{T}{U_{x}}^{-1}M/(N-P)$$ (9)

where ***M*** is a vector of the $\left(y_{i}^{\prime }-y_{i}\right)$ terms shown in Eq. (8).

We can now calculate the value for the quantity *y_k_* at wavelength *λ_k_* through Eq. (7) and its uncertainty through Eq. (2),

$$u^{2}(y_{k})={\left(\frac{\partial f}{\partial a_{i}}|\lambda_{k}\right)}^{T}U_{a}\left(\frac{\partial f}{\partial a_{i}}|\lambda_{k}\right).$$ (10)

Equation (10) is the uncertainty propagated from the input, independent of the quality of the fit. The “goodness” of the fit is represented by the value of *χ*^2^ of Eq. (9), in which the (*N–P*) term is the number of degrees of freedom. For a set of data well-described by the chosen fitting function, *χ*^2^ ≈ 1 [7]; it is a measure of the ratio of the variance of the fit to that of the input values. We can include uncertainty due to the choice of fitting function by scaling the variance calculated in Eq. (10) as

$$u^{2}(y_{k})=\left(1+\chi^{2}\right){\left(\frac{\partial f}{\partial a_{i}}|\lambda_{k}\right)}^{T}U_{a}\left(\frac{\partial f}{\partial a_{i}}|\lambda_{k}\right).$$ (11)

The covariance between any two calculated values *y_k_*. *y_m_* at wavelengths *λ_k_*, *λ_m_* is given from Eq. (5) similarly scaled as

$$u^{2}(y_{k},y_{m})=\left(1+\chi^{2}\right){\left(\frac{\partial f}{\partial a_{i}}|\lambda_{k}\right)}^{T}U_{a}\left(\frac{\partial f}{\partial a_{i}}|\lambda_{m}\right).$$ (12)

Equations (11) and (12) provide the complete variance-covariance matrix for values of a spectral distribution obtained by fitting.

### 2.3 Interpolation

Where data are available at sufficient points to define a distribution known (or suspected) to be smooth, direct interpolation is an alternative to fitting. This is discussed in detail elsewhere [8]. Interpolation provides a mathematical means of estimating new values from a set of input values. Sensitivity coefficients for the dependence of the new values on the input values can be estimated either numerically or by differentiation of the relationship defining them in terms of the original values. Hence uncertainties in the new values, and correlations between them, can be propagated through Eq. (5).

### 2.4 Propagation of Covariance Through a Spectral Transfer

Once reference standards are established, spectral comparison is used to transfer values to secondary or working standards, and similarly from these to particular artifacts. In general terms the transfer process can be written as

$$Q_{i}=t_{i}R_{i}$$ (13)

where *Q_i_* represents the transferred spectral quantity at the *i*th wavelength, *R_i_* is the reference value and *t_i_* is the transfer factor. Provided there is no correlation between the transfer measurement and method used to obtain the reference value, the relative uncertainty in the value of the transferred quantity is given by

$$u_{\text{rel}}^{2}(Q_{i})=u_{\text{rel}}^{2}(t_{i})+u_{\text{rel}}^{2}(R_{i})$$ (14)

### 2.4.1 Uncorrelated Transfer Measurements

If systematic errors and hence correlations in the transfer measurements at different wavelengths are negligible, the covariance between two values *Q_i_*, *Q_j_* is given by

$$\begin{array}{l} u(Q_{i},Q_{j})=\frac{\partial Q_{i}}{\partial R_{i}}\frac{\partial Q_{j}}{\partial R_{j}}u(R_{i},R_{j})\\ =t_{i}t_{j}u(R_{i},R_{j}). \end{array}$$ (15)

The relative covariance between the values *Q_i_*, *Q_j_* remains the same as that between the values *R_i_*, *R_j_* for *i* ≠ *j*. The variance of both *Q_i_* and *Q_j_* is increased [through Eq. (14)] and it is readily shown that the correlation coefficient between the spectral values *Q_i_*, *Q_j_*, defined in Eq. (6), is given by

$$r(Q_{i},Q_{j})=\frac{r(R_{i},R_{j})}{\sqrt{\left[1+\frac{{u_{\text{rel}}}^{2}(t_{i})}{{u_{\text{rel}}}^{2}(R_{i})}\right]\left[1+\frac{{u_{\text{rel}}}^{2}(t_{j})}{{u_{\text{rel}}}^{2}(R_{j})}\right]}},$$ (16)

showing that as the transfer uncertainty increases in each comparison stage, the correlation between pairs of spectral values is decreased.

### 2.4.2 Correlated Transfer Measurements

Where the transfer measurements are themselves correlated between wavelengths, Eq. (15) becomes

$$\begin{array}{l} u(Q_{i},Q_{j})=\frac{\partial Q_{i}}{\partial R_{i}}\frac{\partial Q_{j}}{\partial R_{j}}u(R_{i},R_{j})+\frac{\partial Q_{i}}{\partial t_{i}}\frac{\partial Q_{j}}{\partial t_{j}}u(t_{i},t_{j})\\ =t_{i}t_{j}u(R_{i},R_{j})+R_{i}R_{j}u(t_{i},t_{j}). \end{array}$$ (17)

The relative covariance propagates in a manner analogous to Eq. (14),

$$u_{\text{rel}}(Q_{i},Q_{j})=u_{\text{rel}}(t_{i},t_{j})+u_{\text{rel}}(R_{i},R_{j}).$$ (18)

The correlation coefficient between the spectral values *Q_i_*, *Q_j_* is given by

$$r(Q_{i},Q_{j})=\frac{\frac{u_{\text{rel}}(t_{i})u_{\text{rel}}(t_{j})}{u_{\text{rel}}(R_{i})u_{\text{rel}}(R_{j})}r(t_{i},t_{j})+r(R_{i},R_{j})}{\sqrt{\left[1+\frac{{u_{\text{rel}}}^{2}(t_{i})}{{u_{\text{rel}}}^{2}(R_{i})}\right]\left[1+\frac{{u_{\text{rel}}}^{2}(t_{j})}{{u_{\text{rel}}}^{2}(R_{j})}\right]}}.$$ (19)

Equation (18) extends to any series of multiplicative factors contributing to the transfer. The individual uncertainties may be multiplied to determine the covariance, then added in the relative form of Eq. (18) to determine the total relative covariance.

Where a factor depends upon a single component, the magnitude of the correlation component for that factor is unity. As an example, consider a measurement where the gain of an amplifier used in the recording path for the test artifact drifts in a linear fashion as the wavelength range is stepped during a spectral transfer, but the amplifier used in the reference artifact path is stable. Then the transfer ratio contains a multiplying term

$$t_{i}=1+k(\lambda_{i}-\lambda_{1})$$ (20)

where *k* is the relative change in the gain during the measurement. The covariance between wavelengths for this factor is

$$\begin{array}{l} u(t_{i},t_{j})=\frac{\partial t_{i}}{\partial k}\frac{\partial t_{j}}{\partial k}u^{2}(k)\\ =(\lambda_{i}-\lambda_{1})(\lambda_{j}-\lambda_{1})u^{2}(k). \end{array}$$ (21)

The correlation coefficient between wavelengths for this factor is

$$\begin{array}{l} r(t_{i},t_{j})=\frac{u(t_{i},t_{j})}{u(t_{i})u(t_{j})}\\ =\frac{\left(\lambda_{i}-\lambda_{1})(\lambda_{j}-\lambda_{1})u^{2}(k\right)}{\left(\lambda_{i}-\lambda_{1})u(k)(\lambda_{j}-\lambda_{1})u(k\right)}\\ =+1. \end{array}$$ (22)

and the contribution to the relative covariance is

$$u_{\text{rel}}(t_{i},t_{j})=\frac{u(t_{i})u(t_{j})}{t_{i}t_{j}}.$$ (23)

If all of the correlation coefficients for this term are +1, Eq. (1) shows that the contribution of this effect to the uncertainty of a linear combination of the values at different wavelengths can be found by adding linearly the uncertainties at each wavelength, weighted by the appropriate sensitivity coefficient.

Caution must be applied in using linear sums (or differences) for combining uncertainty components over wavelength where there is a single influence factor, because the sensitivity coefficients (the derivatives) of Eq. (21) may change sign through the spectrum. For example, consider transfer calibration of the spectral responsivity of a detector where the wavelength setting of a monochromator has a fixed offset *Δ* from the true value at which the reference detector responsivity *R*_0_ is known and the test detector responsivity *R* is required. At each wavelength *λ*, the transfer term of Eq. (13) can be written as

$$t_{\lambda +\Delta }=\frac{GS_{\lambda +\Delta }R_{\lambda +\Delta }}{S_{\lambda +\Delta }R_{0,\lambda +\Delta }}$$ (24)

where *S_λ_* is the spectral power incident on the detector and *G* represents amplifier gain differences.

Now

$$R_{\lambda +\Delta }=R_{\lambda }(1+\frac{1}{R_{\lambda }}\frac{dR_{\lambda }}{d\lambda }\Delta )$$ (25)

and it follows that to first order,

$$t_{\lambda +\Delta }=t_{\lambda }\left(1+\left[\frac{1}{R_{\lambda }}\frac{dR_{\lambda }}{d\lambda }-\frac{1}{R_{0,\lambda }}\frac{dR_{0,\lambda }}{d\lambda }\right]\Delta \right).$$ (26)

The covariance between values of the transfer at wavelengths denoted by *i* and *j* is given by

$$u(t_{i+\Delta },t_{j+\Delta })=\frac{\partial t_{i+\Delta }}{\partial \Delta }\frac{\partial t_{j+\Delta }}{\partial \Delta }u^{2}(\Delta ).$$ (27)

Substituting Eq. (26) gives

$$\begin{array}{l} u(t_{i},t_{j})=t_{i}t_{j}\left[\frac{1}{R_{i}}\frac{dR_{i}}{d\lambda }-\frac{1}{R_{0,i}}\frac{dR_{0,i}}{d\lambda }\right]\\ \;\left[\frac{1}{R_{j}}\frac{dR_{j}}{d\lambda }-\frac{1}{R_{0,j}}\frac{dR_{0,j}}{d\lambda }\right]u^{2}(\Delta ). \end{array}$$ (28)

and the correlation coefficients for t given by

$$r(t_{i},t_{j})=\frac{u(t_{i},t_{j})}{\sqrt{u^{2}(t_{i})u^{2}(t_{j})}}$$ (29)

may be +1 or −1 depending on the signs of the relative slopes in Eq. (28). A wavelength offset of 0 nm (± 0.5 nm), caused by uneven illumination of the grating of a monochromator, is sufficient to produce an uncertainty of order 0.1 % in the calibration of the luminous response of a photometer if the reference detector is a silicon trap.

In practice, it is convenient to propagate uncertainties through a measurement process using Eq. (14) and Eq. (18) and to calculate correlation coefficients at any stage using Eq. (6) in its relative form.

## 3. NIST Primary Standard for Spectral Responsivity

Visible and near-visible spectral resonsivity measurements at NIST are based on the High Accuracy Cryogenic Radiometer (HACR). Realization of the spectral responsivity scale at different wavelengths is described in detail in Gentile et al. [9] The responsivity of a silicon trap-detector measured at 9 wavelengths in the range 406 nm to 920 nm was fitted to a model of silicon quantum efficiency; the fitted parameters were then used to calculate the responsivity at any wavelength within the measurement range. The analysis process used by Gentile et al. is repeated here, but with correlations included in the uncertainty propagation and estimated between responsivity values at different wavelengths.

Gentile et al. determined the oxide thickness of the silicon photodiodes in the trap detector to be 28.05 nm (± 0.42 nm) by fitting measured values of reflectance at 3 wavelengths, as an average over two traps and where the uncertainty in the thickness was expanded to include a lack-of-fit component. This thickness was used to calculate the reflectance of the trap *R*_t_ at any wavelength from knowledge of the complex refractive indices of silicon and silicon dioxide [10]. The uncertainty in each value of *R*_t_ is calculated from the sensitivity coefficient of reflectance vs thickness (determined numerically). All of the reflectance values are fully correlated, depending only on the oxide thickness. All of the sensitivity coefficients are negative in the wavelength range covered here, and hence the correlation coefficient between wavelengths is +1, from which we can calculate the covariance matrix for *R*_t_.

The responsivity measurements from HACR are simply scaled to external quantum efficiency *n*_e_ [9]. These measurements are correlated, through the wavelength-independent components in the measurement of optical power with the cryogenic radiometer. Each value of *n*_e_ can be written in the form

$$n_{e,i}=m_{i}m$$ (30)

where *i* denotes wavelength, *m_i_* is the combined independent measurement components and *m* is the common component. The covariance between values at wavelengths *i, j* is

$$\begin{array}{l} u(n_{e,i},n_{e,j})=\frac{\partial n_{e,i}}{\partial m}\frac{\partial n_{e,j}}{\partial m}u^{2}(m)\\ =m_{i}m_{j}u^{2}(m). \end{array}$$ (31)

The correlation coefficient is given by

$$\begin{array}{l} r(n_{e,i},n_{e,j})=\frac{m_{i}m_{j}u^{2}(m)}{u(n_{e,i})u(n_{e,j})}\\ =\frac{u_{\text{rel}}^{2}(m)}{u_{\text{rel}}(n_{e,i})u_{\text{rel}}(n_{e,j})} \end{array}$$ (32)

and it is estimated from the values given by Gentile et al. in their Table 2. The total uncertainties from that table are reproduced here in Table 1, along with the common uncertainty component at each wavelength and the correlation coefficients for the external quantum efficiency derived through Eq. (32).

The internal quantum efficiency at each wavelength is given from *n*_e_ and the reflectance as

$$n_{i}=\frac{n_{e}}{1-R_{t}}.$$ (33)

Measurements of *n*_e_ and *R*_t_ are uncorrelated and it follows that the covariance between values of *n_i_* at different wavelengths is given by

$$u(n_{i,i},n_{i,j})=\frac{u(n_{e,i},n_{e,j})}{\left(1-R_{t,i})(1-R_{t,j}\right)}+\frac{n_{e,i},n_{e,j}u(R_{t,i},R_{t,j})}{{\left(1-R_{t,i}\right)}^{2}{\left(1-R_{t,j}\right)}^{2}}.$$ (34)

Uncertainties and correlation coefficients for *n_i_* are given in Table 2. It is only at the shortest wavelengths that the uncertainty of the reflectance value is sufficiently significant to modify the corresponding values for *n*_e_.

The values of the internal quantum efficiency were fitted to the model discussed by Gentile et al.,

$$n_{i}=P_{f}+\frac{1-P_{f}}{\alpha T}\left(1-e^{-\alpha T}\right)-\frac{1-P_{r}}{\alpha (D-T)}\left(e^{-\alpha T}-e^{-\alpha D}\right)$$ (35)

where *α* is the wavelength-dependent absorption coefficient of silicon and the parameters *P*_f_, *P*_r_, *T*, and *D* can be related to the structure of the silicon photodiodes used in the trap detector. The data for the internal quantum efficiency were first fitted unweighted, then weighted using the generalized Newton-Raphson technique of Woeger [6]. In this case, weighting makes little difference to the parameter values as all the values of the quantum efficiency are similar and not greatly different from unity.

Table 3 shows the values of the parameters for both fits, and the uncertainties propagated through the weighted fit from the uncertainties and correlations of the input data. It can be seen that the values of (*P*_f_, *T*) and (*P*_r_, *D*) are strongly correlated to each other, but not to values in the other bracketed set. The value of *χ*^2^ was 0.13, indicating a good fit of the data to the model. Including this value in the subsequent uncertainty calculations, as described above, accounts for variations in the model in part due to uncertainty in the values of absorption coefficient as a function of wavelength (in turn dependent on the complex part of the refractive index of silicon).

Evaluating derivatives of Eq. (35) with respect to the parameters with the value of α for a given wavelength gives the sensitivity coefficients required for the propagation of uncertainties for and correlations between values of internal quantum efficiency *n_i_*; similarly uncertainties and correlations for values of trap reflectance can be propagated through the SiO-Si model. The values for elements of the variance-covariance matrix for external quantum yield *n*_e_ are then given by

$$u(n_{e,i},n_{e,j})=(1-R_{t,i})(1-R_{t,j})u(n_{i,i},n_{i,j})+n_{i,i}n_{i,j}u(R_{t,i},R_{t,j}).$$ (36)

**Fig. 1:**
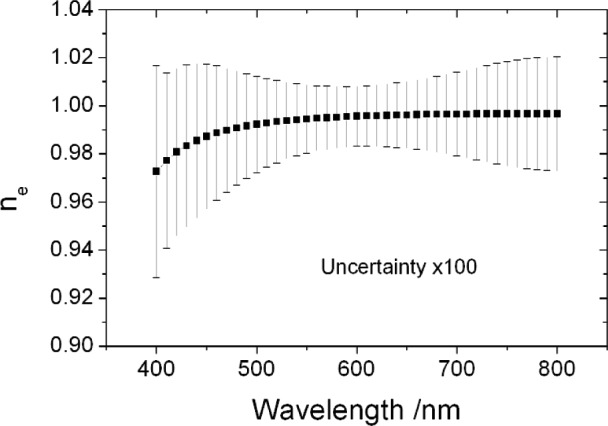
External quantum efficiency *n*_e_ and its relative standard uncertainty (×100) for the visible spectral range propagated from HACR measurements on trap T2.

**Fig. 2:**
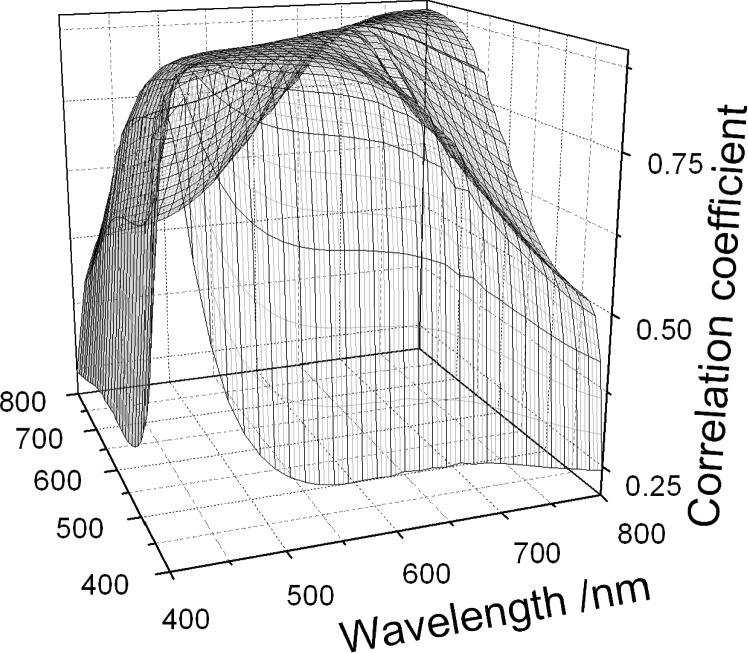
Surface of correlation coefficients for the external quantum efficiency values and uncertainties of Fig. 1. These same correlations apply to the primary spectral responsivity values.

Figure 1 shows *n*_e_ and its standard uncertainty for trap T2 in the visible spectral range; this detector is one of two that carry the primary NIST spectral responsivity scales for this region. Relative uncertainties in responsivity, and the correlation coefficients between them, are identical to those of the external quantum efficiency. Figure 2 is a contour plot of the correlations between the values at different wavelengths (the unity values of self-correlation have been removed). It can be seen that correlations between near-neighbors are strong, and that the correlations vary considerably over the range. Accurate estimation of uncertainty values of subsequent calculations that combine these measurements needs these partial correlations to be taken into account.

## 4. Working Standards of Spectral Responsivity

The NIST spectral comparator facility is used to transfer calibrations from the trap detectors to working standards, as described in detail in Ref. [3]. Table 7.1 of Ref. [3] lists the uncertainty components for the working standard. These values were used to generate data for the transfer alone. The original components for the measurement procedure itself, the digital voltmeter measurements, wavelength setting (assumed random), stray light and bandwidth effects were treated as uncorrelated at the one wavelength and summed in quadrature. Cubic-spline interpolation was used to interpolate the sum to non-table wavelengths. The amplifier gain components were assumed fully correlated at the one wavelength because they are usually measured in the one system dominated by systematic errors with negligible random uncertainty. As these appear in the measurement equation as a ratio, the correlation coefficient is −1 and the net contribution to the total uncertainty of a responsivity expressed as a photo-current is zero.

Two trap detectors were used to transfer the primary reference to the NIST working standards. The systematic errors giving rise to the common uncertainty components of Table 1 are not reduced by this process, but the uncorrelated components are reduced by a factor of $\sqrt{2}$. Hence the calculation beginning in Sec. 3 to this point was repeated with the data shown in Table 4, in which the random components have been averaged. It should be noted that while this averaging reduces the uncertainty in external quantum efficiency, it increases the correlation between wavelengths.

**Fig. 3:**
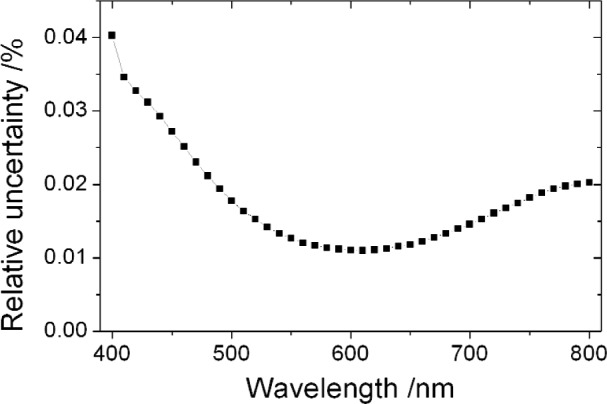
Relative standard uncertainty for the responsivity of a photodetector calibrated against the NIST working standards in the spectral comparator facility.

Client calibrations, including NIST internal calibrations, involve application of a second transfer, for which the uncertainty is small compared to that of the primary reference. The uncertainty after the second transfer is shown in Fig. 3. The correlation between wavelengths after the transfer is little different from that shown in Fig. 2. The main effect of the averaging is to raise the floor value of the correlation coefficients by the order of 0.02. If the reported measurement is a voltage responsivity, the amplifier uncertainty of 0.04 % must be included in the uncertainty propagation. In such a case, the gain measurements are correlated between wavelengths; the correlation can be handled as in Sec. 2.4.2.

An estimate is also made of the uncertainty due to long-term drift in the values of the working standards. The drift values quoted in Ref. [3] are mostly due to short-term temperature variations. The temperature coefficients for a silicon photodiode [11] are all positive, and so the uncertainty component will be fully correlated with a coefficient of +1. It is expected that most systematic effects affecting the long-term drift, such as dust accumulation, will be fully correlated with a coefficient of +1. Uncertainty due to this drift component is approximately equal to the sum of all other non-gain components at visible wavelengths.

## 5. NIST Primary Standard of Spectral Irradiance

The NIST standard of spectral irradiance is a black-body radiator whose operating temperature is calculated from a measurement of irradiance integrated over a band of wavelengths by a filter radiometer, whose responsivity is calibrated against the working standards of spectral responsivity. The filter radiometer response is a close approximation of the luminous efficacy function *V*(*λ*); it was measured at 5 nm intervals with 4 nm resolution. Uncertainty for a *V*(*λ*) filter response was propagated through the chain above. As there was a significant time delay between establishment of the responsivity working standards and the calibration of the filter radiometer, the long-term drift component [3] was added to its uncertainty, fully-correlated between wavelengths.

The procedure to determine spectral irradiance is described elsewhere [12]. The measurement equation for the photocurrent i of the photometer can be written in the form

$$i=k\sum S_{n}L_{n}(T)$$ (37)

where *L_n_* is the spectral radiance of a black-body at wavelength *λ_n_* for temperature *T*,

$$k=\frac{G\;\pi \;r_{\text{BB}}^{2}\;\pi \;r^{2}\;(1+\delta )}{D^{2}}\varepsilon \;\Delta \lambda$$ (38)

is a collection of the geometric terms, the emissivity *ε* of the black-body and Δ*λ*, the wavelength interval (assumed regular) of the spectral responsivity values *S_n_*. Sensitivity components for the temperature uncertainty in terms of the input quantities *i*, *k*, and *S_n_* can be found by implicit differentiation of the zero-valued function

$$f=i/k-\sum_{n}S_{n}L_{n}(T)$$ (39)

as

$$\frac{\partial T}{\partial i}=-\frac{1}{k}/\frac{\partial f}{\partial T},$$ (40)

$$\frac{\partial T}{\partial k}=-\frac{i}{k^{2}}/\frac{\partial f}{\partial T},$$ (41)

and

$$\frac{\partial T}{\partial S_{n}}=L_{n}(T)/\frac{\partial f}{\partial T},$$ (42)

where

$$\frac{\partial f}{\partial T}=-\sum_{n}S_{n}\frac{\partial L_{n}(T)}{\partial T}.$$ (43)

Hence the uncertainty in temperature is given by

$$\begin{array}{l} u^{2}(T)={\left(\frac{\partial T}{\partial i}\right)}^{2}u^{2}(i)+{\left(\frac{\partial T}{\partial k}\right)}^{2}u^{2}(k)\\ \;+\sum_{m}{\left(\frac{\partial T}{\partial S_{m}}\right)}^{2}u^{2}(S_{m})+\sum_{m}\sum_{n\neq m}\frac{\partial T}{\partial S_{m}}\frac{\partial T}{\partial S_{n}}u(S_{m},S_{n}) \end{array}$$ (44)

where *u* (*S_m_*, *S_n_*) is the covariance between *S_m_* and *S_n_*.

The standard uncertainty in temperature for the spectral measurement alone, propagated through the calibration chain described above, is 0.052 K. With substitution of the derivatives, the first two terms of Eq. (44) can be written in terms of the relative uncertainties in *i* and *k* as

$$u(T)=\left(\sum_{n}S_{n}L_{n}(T)/\sum_{n}S_{n}\frac{\partial L_{n}(T)}{\partial T}\right)\sqrt{{u_{\text{rel}}}^{2}(i)+{u_{\text{rel}}}^{2}(k)}.$$ (45)

The multiplying factor in Eq. (45) has a value of 344 K for a *V*(*λ*) filter responsivity and a temperature of 2950 K, equivalent to an effective wavelength of 569 nm. The relative standard uncertainty for the *i* and *k* terms combined is 0.019 %, leading to an uncertainty in temperature of 0.065 K. The multiplying factor also applies to the estimated uncertainty of 0.0001 [13] in the emissivity of the black-body, for which the temperature uncertainty is 0.034 K. Combining all these terms leads to a standard uncertainty in the temperature of the blackbody of 0.090 K.

The spectral irradiance of the blackbody at wavelength *λ_n_* is then given by

$$E_{n}=\frac{\pi \;r_{\text{BB}}^{2}\;\pi \;r^{2}\;(1+\delta )}{D^{2}}\varepsilon \;L_{n}(T).$$ (46)

Given the relative standard uncertainties of 0.0001 in emissivity, 0.05 % for the combined geometric terms for measurements with the Fully Automated Spectro-radiometric Calibrations facility (FASCAL) [12] and the uncertainty of 0.090 K in temperature, we can calculate the uncertainty in spectral irradiance of the black-body for the FASCAL spectral range of 250 nm to 2400 nm, at an operating temperature of 2950 K. This calculation assumes that the spectral emissivity of the black-body, determined essentially in the visible spectral range, is constant over the full spectral range. The assumption is supported by spectral comparison of this variable-temperature black-body with a fixed-point black-body [13]. All of these values are fully correlated, with a correlation coefficient of +1 independent of wavelength, as the radiance of a black-body increases at all wavelengths as the temperature increases and the temperature is the sole-influence parameter between wavelengths.

## 6. Working Standards of Spectral Irradiance

Spectral irradiance working standards are FEL-type lamps, calibrated against the variable-temperature black-body in the NIST FASCAL facility [12]. Uncertainty components for this transfer have been listed in detail in Ref. [12]. Table 5 lists the components, whether they are correlated between wavelengths, the relative standard uncertainty at a wavelength of 655 nm and whether they are wavelength dependent. Wavelength uncertainty (assumed random, with a standard uncertainty of 0.05 nm in the set value) has two effects. The first is direct, in determining the value of the spectral irradiance at the desired setting. The second applies only to the relative shift in spectral irradiance between the black-body source at a temperature of 2950 K and that of the lamp, typically with a distribution temperature near 3200 K; this second effect is much reduced compared to the first. The black-body temporal stability component is correlated between wavelengths because it represents a slow temperature drift during measurements that are sequential with wavelength; as a minor component, it is taken as fully correlated here. By contrast, the lamp current may fluctuate on a much shorter time scale and so its effect is uncorrelated between wavelengths.

**Fig. 4:**
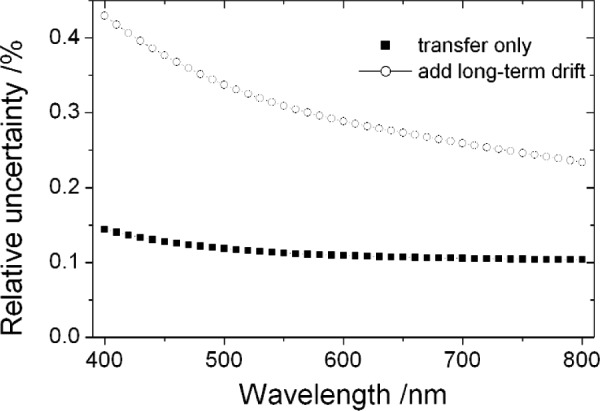
Relative standard uncertainty of the spectral irradiance working standards in the visible wavelength range, with and without the long-term drift component.

The lamp standards drift with use. This drift, generally inversely proportional to wavelength [14], is correlated between wavelengths and it is the dominant uncertainty component. Figure 4 shows the relative standard uncertainty for the spectral irradiance of the working standards in the visible spectral range, with and without the drift component included. Correlation coefficients between spectral irradiance values in the visible spectral region range in value from 0.35 to 0.50. Adding in the long-term drift raises these values to the range 0.90 to 0.93.

### 6.1 Interpolation of NIST Standards of Spectral Irradiance

**Fig. 5:**
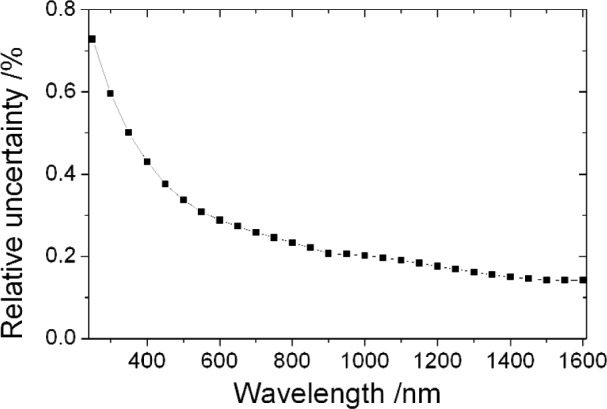
Relative standard uncertainty of the spectral irradiance working standards over the 250 nm to 1600 nm wavelength range of a typical calibration.

**Fig. 6:**
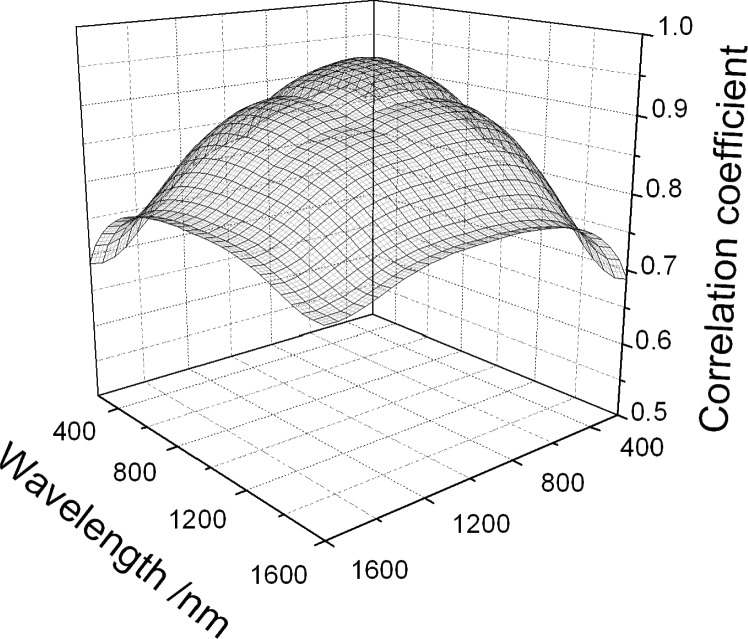
Correlation coefficients for the spectral irradiance working standards between wavelengths over the 250 nm to 1600 nm wavelength range of a typical calibration.

Most NIST calibrations of spectral irradiance are provided at 26 discrete wavelengths in the range 250 nm to 1600 nm [1]. Fig. 5 shows relative standard uncertainties and Fig. 6 shows the correlation coefficients for this range, calculated as in the previous section. NIST procedure is to interpolate these data using a two-stage fit representing a black-body distribution modified by a degree-5 polynomial representing emissivity, preferably in two spectral ranges [1]. Problems of fitting the data have been previously addressed [15]. An alternative method is to interpolate directly, using for example a cubic-spline [8]. In either case, uncertainties should be propagated through the process used, including correlations between the measured values. Neither interpolation method will provide accurate values in the region of known spectral absorption or emission lines.

**Figure 7:**
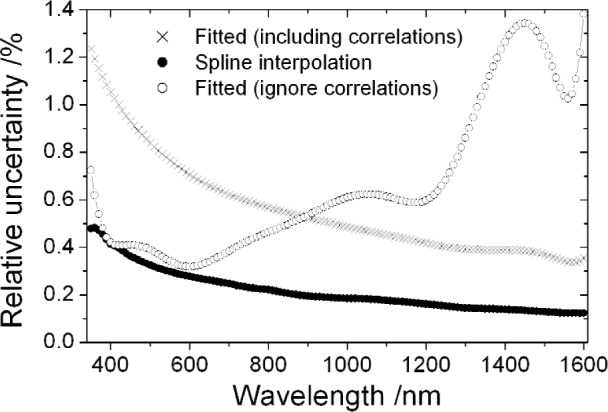
Relative standard uncertainty for interpolated values of spectral irradiance of a calibrated lamp. Fitting is by the recommended NIST SP250-20 method, with correlations included and ignored.

Figure 7 shows uncertainties propagated from the lamp distribution given in Ref. [1] (with the value at 555 nm corrected to the true value of 0.102 W · m^−2^ · nm^−1^) for both cases, using the uncertainties and correlations propagated as above. Only the upper spectral range of 350 nm to 1600 nm is considered here. The cubic-spline interpolation reproduces the uncertainty at the input points. The higher-values of uncertainty for the fitted values arise because the reduced value of chi-squared is ≈ 5. (The relatively poor fit was masked in previous calculations by the much larger uncertainty assigned to the black-body temperature determination.) The uncertainty distribution for both cases follows the expectation for a thermal source. Figure 7 also shows the uncertainty distribution calculated from a conventional weighted fit that does not take into account the correlations between the input points. While the average value through the spectral range is similar to the fitting including correlations, the distribution over wavelength is not one expected on physical grounds, showing the importance of including the correlations.

**Fig. 8:**
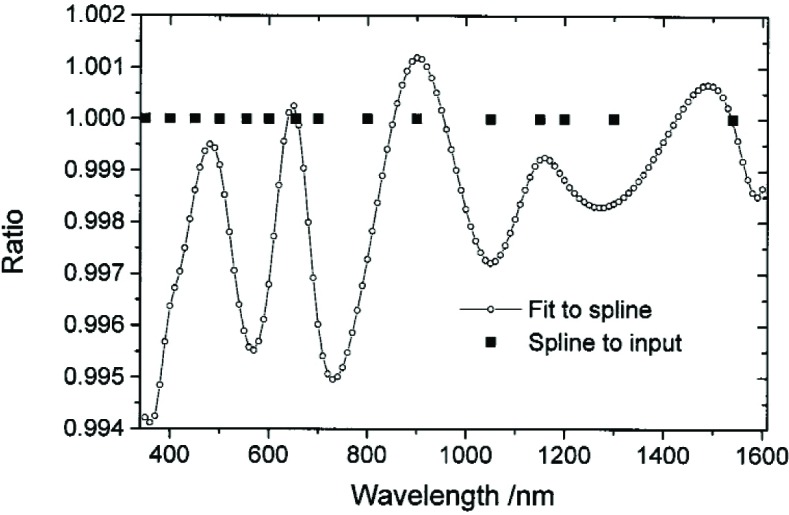
Ratio of values calculated by fitting to those by spline interpolation. The points *spline to input* show the position of the input values.

The fitting process, particularly when made in two stages, has a low number of degrees of freedom, with the attendant problems of high residuals near the endpoints of the range if checked against data on a finer spacing. The cubic-spline interpolation of smooth data is not expected to show such structure. Figure 8 shows the ratio of interpolated values obtained by fitting (including correlations) to those obtained by a cubic-spline method. The structure shown is an artifact typical of polynomial fitting. Spline interpolation reproduces the input values, as shown in the figure. Fitting has the ability to smooth through random variations in the input data, provided the fit function has a good physical basis. It is recommended that cubic-spline interpolation be used to interpolate the calibrated spectral irradiance values to intermediate wavelengths, with the caveat that regions of known line features should be avoided.

Correlation coefficients for spline-interpolated values at different wavelengths in the important visible region are in the range 0.92 to 1.0, reflecting the highly correlated input data. Given this fact, uncertainty estimates for combined spectral data may be made by interpolating the data itself, interpolating the quoted uncertainties and then assuming that the interpolated values are fully correlated. This method will remain valid while the long-term drift component dominates the uncertainty.

## 7. NIST Photometric Standards

The results of Sec. 4 can be applied to the integrated response of a photometer. For a true Illuminant A source, a photometer whose *V*(*λ*) response was measured against the NIST spectral responsivity working standards at 5 nm intervals will have a relative standard uncertainty in response of 0.016 %. This is one required component when determining the photometric units [2].

Calibrations are also provided of correlated color temperature (CCT), by comparison with a lamp calibrated against the NIST spectral irradiance standards at a wavelength interval of 5 nm. For an Illuminant A source, i.e. a CCT value of 2856 K, the standard uncertainty in CCT is 0.9 K [16,17]; uncertainties in the intermediate (*u,v*) chromaticity values are (0.00004, 0.00001) with a correlation coefficient of 0.66 between them. For this same source, the uncertainty in distribution temperature is 0.8 K [6]. Further uncertainty is added in the transfer process to the device under test.

## 8. Continuously-Tunable Laser Systems

Laser-based systems continuously tunable in wavelength over a wide spectral range are now being coupled to cryogenic radiometers [18]. Many more wavelengths than the fixed values used with HACR may be used to measure the quantum yield of the transfer trap detectors. This is particularly useful in the wavelength region below 400 nm, where reliable modeling of the quantum yield of a particular set of silicon photodiodes may be difficult [19, 20]. Residuals in the fitting of a particular function can be large compared to the measurement uncertainty, and the silicon properties used to model the surface reflectance loss may not be reliable. In these regions, the new laser systems allow measurements to be taken at closely spaced wavelengths so that reliable interpolation can be made without the need for a physical model. The spectral responsivity of a trap detector can be interpolated directly, varying the wavelength spacing of the data points to suit the rate of change in responsivity with wavelength. The example in Sec. 6 shows that uncertainty propagation through direct interpolation can lead to lower, more realistic uncertainties (but more strongly correlated) than those obtained by fitting.

## 9. Conclusion

Many of the national metrology institutes realizing primary standards in spectral radiometry now trace their reference to a cryogenic radiometer in a way similar to that described here, at least for the visible spectral range. Correlations between values at different wavelengths in the base data may be high. Proper understanding of the uncertainties in calibrated artifacts based on such standards then requires that these correlations be propagated through the calibration chain. Methods of realizing spectral irradiance may differ in detail from those used at NIST, but fitting and interpolation are common and the techniques described here apply.

The examples here lead to generally lower uncertainties than previous estimates. Inter- and intra-laboratory comparisons may then lead to results inconsistent with a given uncertainty analysis. Proper estimation of uncertainties, rather than over-estimation, then leads to the increased probability of detecting systematic effects which may have been overlooked in the original analysis, which in turn leads to a better understanding of the practice of spectral radiometry. Correlations in the spectral quantities between wavelengths are generally partial and may be high; these must then be included in the uncertainty analysis of subsequent radiometric calculations that combine the spectral quantities. One clear outcome of the analysis is that both spectral irradiance values and their uncertainties provided by NIST at visible wavelengths can be simply interpolated for subsequent measurements and uncertainty estimation, with the interpolated values taken as fully correlated.

## Figures and Tables

**Table 1:** Total relative standard uncertainty, common relative standard uncertainty in values of the external quantum efficiency of trap T2 at the measurement wavelengths, and the correlation coefficient between the values at the different wavelengths

Wavelength (nm)	Total uncertainty (%)	Common uncertainty (%)	Correlation coefficients								
406.74	0.027	0.021	1.00	0.56	0.52	0.40	0.37	0.46	0.54	0.23	0.22
441.57	0.028	0.021		1.00	0.51	0.39	0.36	0.45	0.52	0.22	0.21
487.99	0.030	0.021			1.00	0.36	0.34	0.42	0.49	0.21	0.19
514.53	0.039	0.021				1.00	0.26	0.32	0.38	0.16	0.15
532.08	0.042	0.021					1.00	0.30	0.35	0.15	0.14
632.82	0.034	0.021						1.00	0.43	0.18	0.17
769.64	0.029	0.021							1.00	0.22	0.20
828.30	0.027	0.013								1.00	0.22
919.85	0.029	0.013									1.00

**Table 2:** Standard relative uncertainty of the internal quantum efficiency at the measurement wavelengths and correlation coefficients between wavelengths

Wavelength (nm)	Uncertainty (%)	Correlation coefficient								
406.74	0.034	1	0.65	0.54	0.40	0.37	0.42	0.46	0.21	0.19
441.57	0.030		1	0.54	0.41	0.38	0.44	0.50	0.23	0.21
487.99	0.031			1	0.38	0.35	0.42	0.49	0.21	0.20
514.53	0.039				1	0.27	0.33	0.38	0.17	0.15
532.08	0.042					1	0.3	0.35	0.15	0.14
632.82	0.034						1	0.43	0.19	0.17
769.64	0.029							1	0.22	0.20
828.30	0.027								1	0.22
919.85	0.029									1

**Table 3:** Fit parameters, their standard uncertainties and correlations for the silicon photodiode internal quantum efficiency

	Un-weighted fit	Weighted fit	Uncertainty	Correlation coefficient			
*P*_f_	0.9748	0.9744	0.0015	1.00	0.93	0.01	−0.08
*T* (µm)	0.281	0.272	0.029		1.00	0.05	−0.19
*P*_r_	0.99775	0.99773	0.00068			1.00	−0.86
D (µm)	27.9	29.1	17.6				1.00

**Table 4:** Repeat of the quantities of Table 1, averaged over two trap detectors

Wavelength (nm)	Total uncertainty (%)	Common uncertainty (%)	Correlation coefficients								
406.74	0.024	0.021	1.00	0.72	0.69	0.57	0.53	0.63	0.70	0.33	0.31
441.57	0.025	0.021		1.00	0.67	0.55	0.52	0.61	0.69	0.32	0.31
487.99	0.026	0.021			1.00	0.53	0.50	0.59	0.66	0.31	0.29
514.53	0.031	0.021				1.00	0.41	0.48	0.54	0.26	0.24
532.08	0.033	0.021					1.00	0.46	0.51	0.24	0.23
632.82	0.028	0.021						1.00	0.60	0.28	0.27
769.64	0.025	0.021							1.00	0.32	0.30
828.30	0.021	0.013								1.00	0.35
919.85	0.022	0.013									1.00

**Table 5:** Uncertainty components (relative standard uncertainty) for the working standards of spectral irradiance propagated from the black-body (BB)

Component	Uncertainty at 655 nm (%)	Correlated between wavelengths?	Wavelength dependent?
BB irradiance	0.055	Yes	Yes
BB spatial uniformity	0.05	Yes	No
BB temporal stability	0.03	Yes	Yes
Wavelength accuracy (0.05 nm)	0.02	No	Yes
Spectroradio- meter stability	0.05	No	No
Transfer stability	0.05	No	No (below 2300 nm)
Lamp current stability	0.02	No	Yes
